# Does the Directive to Avoid Low Back Flexion Hinder Physical Performance? Examining Isometric Strength in Postures Adopted During Light Mass Lifting

**DOI:** 10.1177/00187208251404836

**Published:** 2025-12-08

**Authors:** Brendan L. Pinto, Tyson A. C. Beach, Jack P. Callaghan

**Affiliations:** 1 8430 University of Waterloo, Canada

**Keywords:** manual materials handling training, spine, instruction, cue, coach

## Abstract

**Objective:**

Observe how instruction to avoid rounding the low back while lifting a relatively light mass impacts isometric lifting strength.

**Background:**

As opposed to manual materials handling training directives recommending whole-body techniques such as a squat lift, targeting specific body regions such as low back curvature, theoretically affords workers greater flexibility to organize the rest of the body to reduce musculoskeletal loading without reducing physical performance. However, providing these directives during sub-maximal tasks may not prompt prioritization of physical performance as individuals self-organize, eventually making the intervention ineffective.

**Methods:**

Forty participants (50% female) lifted a crate with and without the instruction to avoid rounding the low back. Postures at the initiation of crate lifting were replicated to test isometric strength.

**Results:**

At the group-level, instruction decreased low back flexion (*p* < 0.0001) but did not change strength (*p* = 0.862). However, high heterogeneity motivated examining individual responses. Thirty-seven participants (92.5% of the sample) exhibited greater than 40% of their flexion range-of-motion during baseline lifting, a threshold below which passive tissue strain is typically minimized. Yet, 22 participants (55%) were unsuccessful in reducing low back flexion below this threshold with instruction. Independent from these postural response groups, 23 maintained (57.5%), 8 increased (20%) and 9 decreased (22.5%) isometric strength.

**Conclusion:**

On average, physical performance potential was maintained in response to a low back postural directive. However, personalized movement coaching is needed to ensure the desired response for all.

**Application:**

Manual materials handling training should include personalized movement coaching that considers both musculoskeletal loading and performance.

## Introduction

Interventions to regulate movement behaviour are necessary as individuals do not inherently move to minimize or limit musculoskeletal loading which can counteract engineering controls to reduce occupational task demands such as reducing the mass lifted (Faber et al., 2007; McGill, 2009). However, it is vital that modifying movement to manage musculoskeletal loading does not reduce physical performance, as this may challenge acceptability and effectiveness of the intervention. For instance, the poor adherence and ineffectiveness of the manual materials handling training instruction to adopt a squat lift technique has been in part attributed to decreases in physical performance potential such as decreases in strength (Denis et al., 2020; Kumar & Garand, 1992).

As opposed to recommending a single vaguely defined whole-body lifting posture – which might not be suitable for every context – targeting a specific body region, such as low back posture, offers individuals greater flexibility to configure the rest of their body linkage based on personal characteristics to satisfy task objectives (Beach et al., 2018; Becker et al., 2022; Chan et al., 2022; Frost et al., 2015; Grütters et al., 2023; Larson & Brown, 2023; Pinto et al., 2018). In contrast to the questionable impact of the squat lift technique on reducing musculoskeletal loading (van Dieën et al., 1999), avoiding low back flexion can decrease passive tissue strain (Potvin et al., 1991; Zwambag & Brown, 2020), increase the load tolerance of the functional spinal units (Callaghan & McGill, 2001; Gallagher et al., 2005; Gunning et al., 2001; Howarth & Callaghan, 2012; Solomonow, 2012) and maintain the trunk extensor muscles’ lines of action to counteract potentially injurious anterior shear forces (McGill et al., 2000; Pinto et al., 2021). Avoiding repetitive and sustained low back flexion can further evade residual effects from viscoelastic changes that disrupt sensorimotor function (Abboud et al., 2018; Pinto et al., 2021; Sánchez-Zuriaga et al., 2010; Solomonow, 2012) and pain perception (Viggiani & Callaghan, 2021, 2022).

Although targeting low back posture to reduce musculoskeletal loading provides the opportunity to flexibly configure the rest of the body linkage, it is uncertain if individuals will use this flexibility in ways that maintain physical performance. Reducing low back flexion can acutely reduce isometric lifting strength in some individuals, but when the posture of the rest of the body is experimentally controlled (Pinto et al., 2025). It remains unknown if individuals will maintain strength in response to low back posture focused interventions that provide the opportunity to self-organize the rest of the body. This may be particularly relevant when using the traditional directive-based manual materials handling training approach (Denis et al., 2020), as simple instructions to avoid low back flexion during low demand or sub-maximal tasks may not require individuals to prioritize acute force generating capacity as they self-organize. Adopting weaker postures may initiate and accelerate fatigue-related processes to reduce physical performance and the effectiveness of the intervention.

The objective of this study was to observe how instruction to avoid rounding the low back while lifting a relatively light mass impacts isometric lifting strength. Participants lifted a crate without (i.e. baseline) and with the instruction to avoid rounding the low back. Postures adopted at the initiation of the crate lift for each condition (baseline and instruction) were replicated during isometric strength testing. It was hypothesized that individuals would maintain their ability to exert force in response to low back postural instruction during light mass lifting.

## Methods

### Participants

Forty participants (50% female; group mean ± SD age 23 ± 3 years; mass 76.0 ± 15.3 kg; height 1.73 ± 0.08 m) provided written informed consent prior to participation. Participants were eligible to participate if they were between the ages of 18–64, had no skin allergies or sensitivities to alcohol or adhesives and had no self-reported pain or injury within the 6 months prior to participation that prevented performance of daily activities or other health condition preventing participation in physically strenuous activities. This research complied with the tenets of the Declaration of Helsinki and was approved by the University of Waterloo research ethics board.

### Equipment

Optical motion tracking was performed using Optotrak Certus (Northern Digital Inc, Waterloo, ON, Canada). Rigid bodies with a minimum of three kinematic markers were affixed to the hands, upper arms, head, thighs, shanks, feet and on the trunk overlaying the seventh cervical, twelfth thoracic and first sacral vertebrae. Anatomical landmarks of each body segment were then digitized and monitored with respect to each segment’s marker rigid body to create a whole-body rigid link segment model.

Maximal isometric lifting exertions were performed by attempting to vertically lift a handle (length: 40.1 cm; diameter: 3.2 cm) which was secured to a metal bar attached to the ground, in series with a uniaxial force transducer (MLP-1K, Transducer Techniques LLC, Temecula, CA, United States). A kinematic marker was placed at the centre of the handle and on the force transducer. The height of the handle was approximately 39.5 cm from the ground to match the height of the hands when holding a crate that was to be lifted during the experiment.

Kinematic and force transducer data were sampled simultaneously at 40 Hz and 2000 Hz, respectively, using an Optotrak Data Acquisition Unit II and Optotrak System Control Unit controlled by NDI First Principles software (Version 1.2.4, Northern Digital Inc, Waterloo, ON, Canada).

### Experimental Procedures

**Figure 1.**
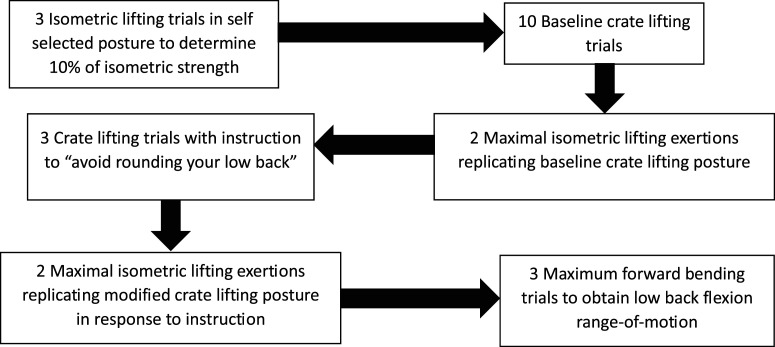
Overview of experimental procedures.

A schematic overview of the experimental procedures is provided in Figure 1. A 10-s relaxed standing trial was used to define the 0° angle of each joint’s posture (Pinto & Callaghan, 2022). A minimum of 3 warm-up isometric lifting trials were performed where participants were instructed to exert 30, 50 and 80% of their perceived maximal effort. This was followed by 3 maximal isometric lifting trials in a self-selected posture.

**Figure 2.**
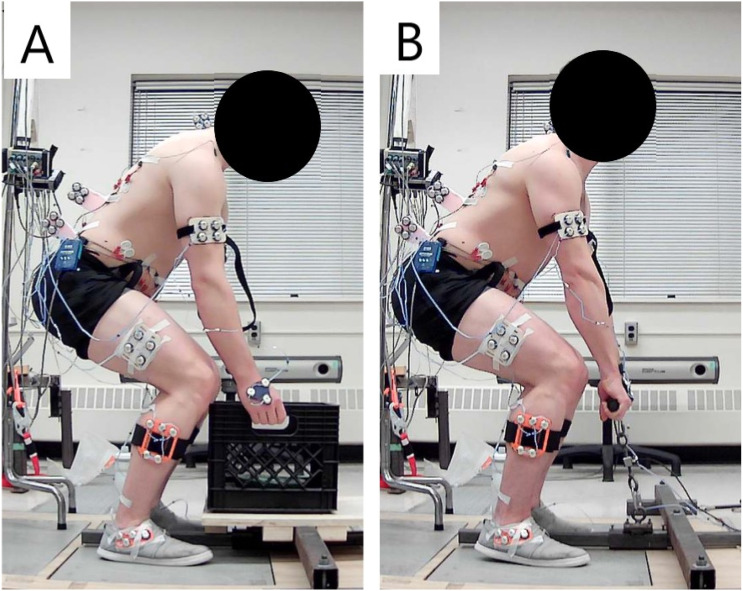
Example of posture replication between the crate and isometric lifting trials. (A) Posture at the initiation of the crate lift which was replicated for the isometric lifts. (B) Participant exerting maximal effort during an isometric lifting trial in the replicated posture.

A crate loaded with mass approximating 10% of the maximum vertical isometric force was placed on a platform so it could lay flat over the metal bar which secured the isometric strength testing handle (Figure 2). Ten separate baseline crate lifts were performed with the instruction ‘to lift naturally and as you regularly would in everyday life’. For each crate lifting trial, participants started from standing, reached and lifted the crate from the platform to a standing position, lowered the crate and finally returned to the standing position without the crate. Ten trials were performed to facilitate a steady state and natural baseline. The position of the crate on the platform was kept consistent across trials.

On the 10th baseline crate lift, participants were instructed to maintain their foot position until the completion of 2 maximal isometric lifting exertions. These maximal exertions were performed in a posture replicating the instant that the participant initiated lifting the crate from the platform. To accomplish this, an image of the sagittal plane of the participant at the instant they lifted the crate from the platform was captured on the 10^th^ baseline crate lifting trial (Figure 2(a)). The isometric strength testing handle matched the height of the crate handles and the attachment point of the handle on the metal bar that secured it to the ground, was aligned with the centre of the base of the crate on the platform. The image of the participant’s lift posture was used to create a silhouette which was overlaid on a live video feed on a computer screen. This screen was set in view of the participant so they could match their posture to the silhouette. The range in peak joint flexion/extension angles of the low back, right hip and right knee across the last 3 baseline crate lifting trials and real-time outputs of these joint angles from the NDI First Principles software (Version 1.2.4, Northern Digital Inc, Waterloo, ON, Canada), were used to verify that participants were within range of the target posture. Once the posture was replicated participants exerted maximal isometric lifting force (Figure 2(b)). Participants were instructed to maintain the setup posture during the maximal exertions. A minimum of 2 min of rest were taken between maximal isometric lifting trials.

Three crate lifting trials were then performed with only the instruction to ‘avoid rounding your lower back’. The researcher (BLP) also briefly visually demonstrated the difference between flexion and extension of the low back to indicate that ‘rounding’ referred to a flexed curvature. No other explanation, description or coaching was given regarding low back posture or how to lift. This was followed by 2 maximal isometric lifting trials replicating the crate lifting posture exhibited during the instruction condition. Conditions were performed in a sequential order to accurately capture baseline behaviour. If crate lifting trials were randomized, performing the instruction condition first would have required participants to potentially modify their natural movement and would have drawn their attention to their movement. This would have interfered with subsequent attempts to produce natural movement behaviour. The isometric strength testing trials were performed immediately after the crate lifting trials for each condition to facilitate replication of the crate lifting postures. During pilot data collections, it was beneficial for participants to maintain their foot position as opposed to replicating it later. Additionally, posture replication was sometimes facilitated by cueing participants to imagine reaching for the crate as they had in the previous crate lifting trial. This cue would have been ineffective if the isometric trials were not performed immediately after the crate lifting trials. Based on these initial observations, performing the isometric strength testing immediately after each crate lifting condition was expected to help overcome any unforeseen participant-specific challenges in posture replication.

Finally, 3 trials of standing maximum low back flexion were performed to normalize the data for each individual to their range-of-motion. Participants were coached on rotating their pelvis and ribcage to achieve maximum low back flexion as they bent their chest towards their thighs. At volitional end range of low back flexion, a 3-s trial was collected during which participants were encouraged to flex their low back further. These trials were performed after the experimental conditions to avoid interfering with participants’ natural response and ability to modify low back posture.

### Post Collection Data Processing

Kinematic data were processed in Visual3D (Version 2022, HAS-Motion, Inc, Kingston, ON, Canada) and filtered with a dual pass low pass Butterworth filter with a 10 Hz cutoff (Kristianslund et al., 2012; Tomescu et al., 2018). Whole-body rigid link segment models adjusted for participants’ height and weight were generated. Low back, right hip and right knee 3D joint angles were calculated relative to relaxed standing, using a flexion/extension, lateral bend and axial twist Cardan angle rotation sequence. These data were imported in the subsequent analysis performed in Python (Spyder IDE, Version 5.3.0, Spyder Project Contributors).

**Figure 3.**
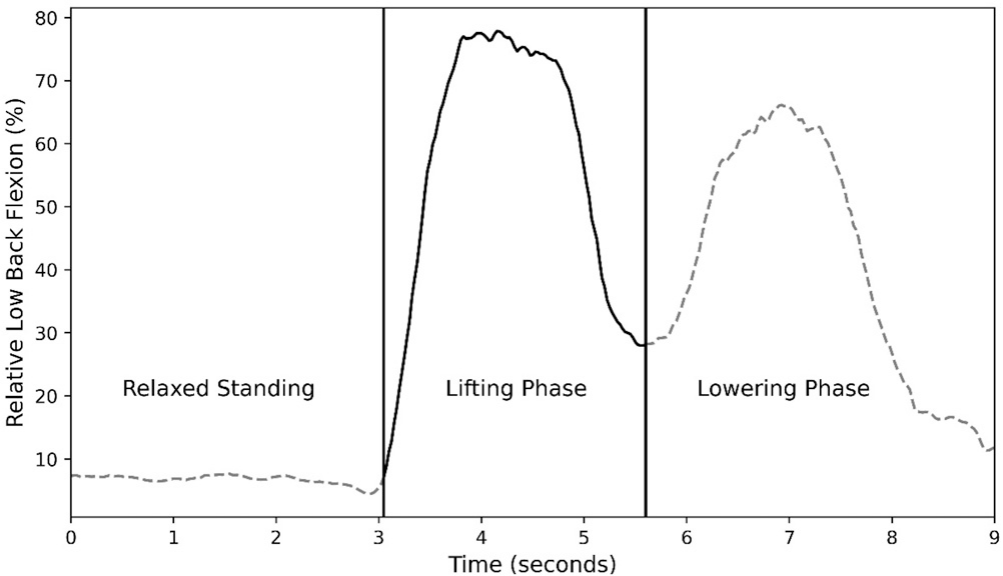
Sample participant data depicting the identified lifting phase plotted using a solid black line. The vertical solid black lines represent the start and end of the lifting phase.

The start and end of the lifting phase of the crate was identified using the first derivative approach and the right hip flexion/extension joint angle (Pinto & Callaghan, 2023; Figure 3). Specifically, the start was defined as the first instant of the longest time period where the first derivative of the right hip flexion/extension angle exceeded 5 times the standard deviation of 1.5 s of relaxed standing at the start of each trial. This marked the onset of a continuous increase in hip flexion as the participant bent towards the ground. The end of the lifting phase was marked as the first instant of the longest duration of flexion velocity, between the time point where the first derivative first reached 60% of maximum extension velocity and the succeeding time point where the first derivative reached a minimum flexion velocity. The maximum low back, right hip and right knee flexion achieved during the identified lifting phase of each trial were extracted and low back flexion was normalized to participants’ low back flexion range-of-motion.

The peak vertical component of the isometric lifting force was estimated by interpolating the unit vector between the handle and force transducer to 2000 Hz and multiplying it by the force recorded by the force transducer.

### Statistical Analysis

Linear mixed effects models were used to analyze the effect of condition (baseline and instruction; the independent variable) on low back posture during crate lifting and isometric lifting strength (dependent variables; lmerTest package, RStudio Version 2023.03.0, The R Foundation, Vienna, Austria). Linear mixed effects models were used to preserve information and statistical power by conserving all the recorded trials, to account for unbalanced design (10 baseline and 3 instruction), to avoid pseudo-replication by accounting for trial dependence and to quantify the heterogeneity in participant responses by estimating intercepts and slopes for each participant. The model consisted of condition (2 levels: baseline or instruction) as the fixed effect with a random slope and a random intercept for each participant. Although the sequential design was not expected to impact the low back posture responses to instruction during crate lifting, it could potentially confound the interpretation of the isometric strength responses due to time-dependent factors such as learning to improve force production in the replicated postures across the four isometric strength trials conducted. Thus, the trial number (1–4) was included as a fixed effect interaction with condition in the model for isometric lifting strength. This adjusted the effect of condition for potential systematic time-dependent changes across sequential trials, assessed the effect of time and determined if the effect of condition was dependent on trial number. Statistical significance was considered if *p* < 0.05.

**Figure 4.**
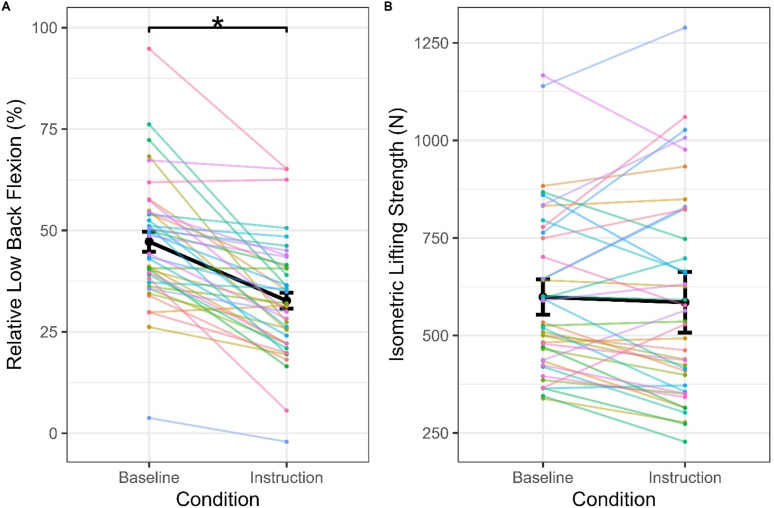
Relative low back flexion (A) and isometric lifting strength (B) across conditions. Estimated marginal means are represented in black with the error bars representing standard error. The estimates for each participant from the linear mixed effects model are depicted by the coloured points and lines, where each colour represents a participant. Significant differences between conditions are indicated by “*”.

A post hoc analysis to classify individual responses was motivated by the results of the linear mixed effects models which indicated that the low back posture and isometric strength responses to the instruction condition greatly varied across participants (Table 1, Figure 4). Responses in low back posture during the crate lift were first classified using personalized confidence intervals that were calculated using the within participant trial-to-trial standard deviation ( ${SD}_{WP}$ ; Swinton et al., 2018):

$$Confidence\;Interval=\sqrt{2}\times 1.96\times {SD}_{WP}$$

This personalized confidence interval was estimated for each condition to describe the range within which each participant’s response was expected fall based on the variability they exhibited, with 95% confidence. The interval was then compared against a threshold of 40% of their low back flexion range-of-motion to classify low back postural responses, as low back passive stiffness and tissue strain are typically minimized within this threshold and exponentially increase beyond it (Beach et al., 2005; Buchman-Pearle et al., 2023; Fewster et al., 2021). It was anticipated that some participants would already exhibit less than 40% of their low back flexion range-of-motion at baseline and would not greatly benefit from further reducing flexion compared to those above this threshold at baseline. Hence, participants were first classified into two groups based on whether the upper-limit of their baseline confidence interval for low back posture was below or above 40% of their flexion range-of-motion. Within these two groups (<40% and >40% baseline low back flexion), participants were classified as successful (S) or unsuccessful (U) at avoiding low back flexion based on whether the upper-limit of their instruction condition confidence interval was below or above 40% of their low back flexion range-of-motion, respectively. Thus, individuals were considered to successfully respond to the instruction and avoid rounding their lower back if their low back posture was likely to be consistently within 40% of their flexion range-of-motion with 95% certainty, based on the variation they exhibited. The confidence intervals were calculated from all 10 trials for the baseline condition and all 3 trials from the instruction condition.

The changes in isometric lifting strength between conditions were classified based on the combined trial-to-trial ${SD}_{WP}$ across conditions. The combined variation across all trials for both conditions were used to acquire a stronger estimate of ${SD}_{WP}$ by subtracting the mean of the two isometric lifting trials within each condition from each trial within the respective condition. This centred the variation within each condition at 0 by removing the difference in magnitude between conditions but maintaining the variation between the trials within each condition. Assuming that the trial-to-trial variation was consistent between conditions, the ${SD}_{WP}$ for isometric lifting strength was calculated across all 4 mean-bias-removed maximum isometric trials. Individual changes in maximum isometric lifting strength were classified as an increase (I), decrease (D) or no change (S) if the difference between baseline and instruction conditions were respectively greater, lesser or within the participant specific confidence interval. The difference between conditions was calculated using the mean of the 2 maximum isometric lifting trials for each condition.

## Results

At the group-level, low back flexion significantly decreased (*p* < 0.0001) in the instruction condition compared to baseline, but isometric lifting strength did not change significantly (*p* = 0.862). There was no effect of trial number (*p* = 0.864) or its interaction with condition (*p* = 0.442) on isometric strength, indicating that the sequential order of the conditions did not systematically influence strength and that the effect of condition did not change over trials. However, the random effects standard deviation from the linear mixed effects models indicated substantial heterogeneity in responses. Specifically, the random effects standard deviation predicted that 11.5% of the population would be expected to exhibit an opposite response to the group-level response in low back flexion, based on a standard normal cumulative distribution. Additionally, the random effects standard deviation was 10 times greater than the group-level response in isometric lifting strength, indicating high between-participant variability in magnitude and direction of isometric strength responses (Table 1). Yet, these estimates represent the expected probability within a population rather than the actual count of individuals within the current sample. Visualizing the data also indicated high heterogeneity and suggested that the group-level responses in low back flexion were driven by large changes in some individuals, whereas others showed small and varying changes (Figure 4). Together this motivated a post hoc analysis to classify individual responses.

**Table 1.** Linear Mixed Effect Model Results for Changes in Low Back Posture and Isometric Lifting Strength Between the Baseline and Instruction Conditions.

	Fixed Effects		Random Effects		
	Intercept ± SE	Estimate (β) ± SE	SD Intercept	SD Estimate	Corr
Relative low back flexion (%)	47.23 ± 2.49	−14.54 ± 1.97*	15.67	12.08	−0.46
Isometric lifting strength (N)	598.47 ± 45.50	−13.52 ± 77.58	215.05	146.08	0.07

The intercept represents the value at baseline, whereas the estimate is the change from baseline during the instruction condition. The random effects Corr represents the correlation between each participant’s intercept (i.e. baseline) and estimate (i.e. change from baseline).

*p < 0.05.

**Figure 5.**
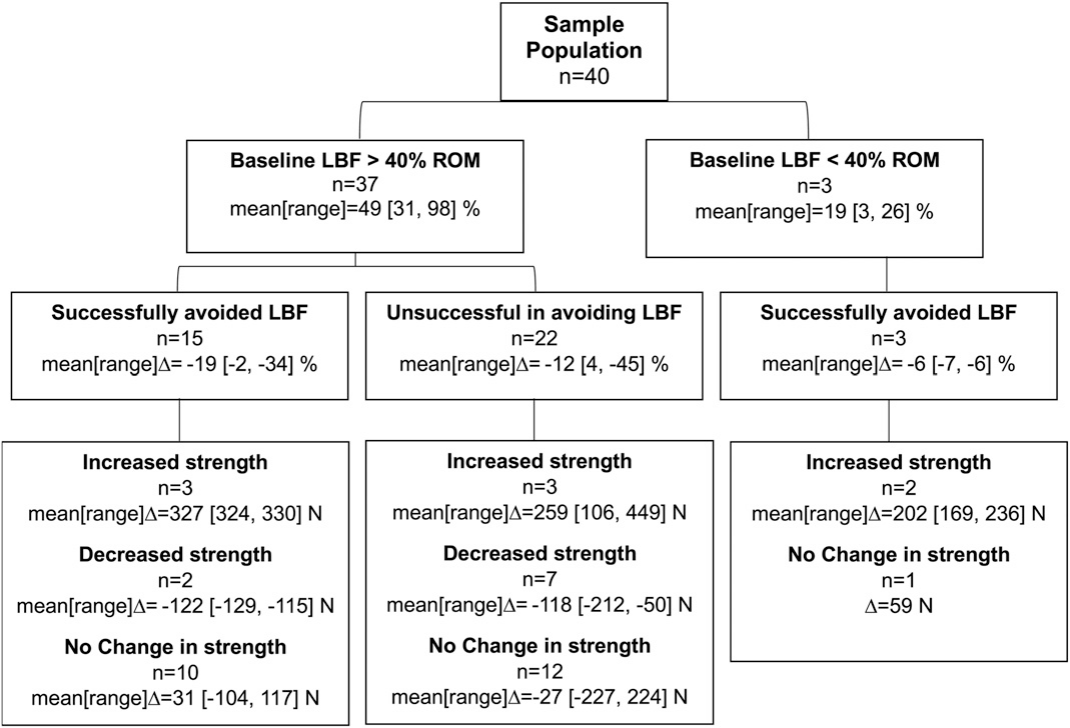
Classification of low back flexion (LBF) and isometric lifting strength responses. The mean [range] at baseline and the mean[range]Δ between the instruction and baseline conditions represent values across participants, where negative Δ values represent a decrease from baseline. Note that the posture and strength values are the means within each condition, whereas the response classification was based on within participant variation. ROM refers to flexion range-of-motion.

**Figure 6.**
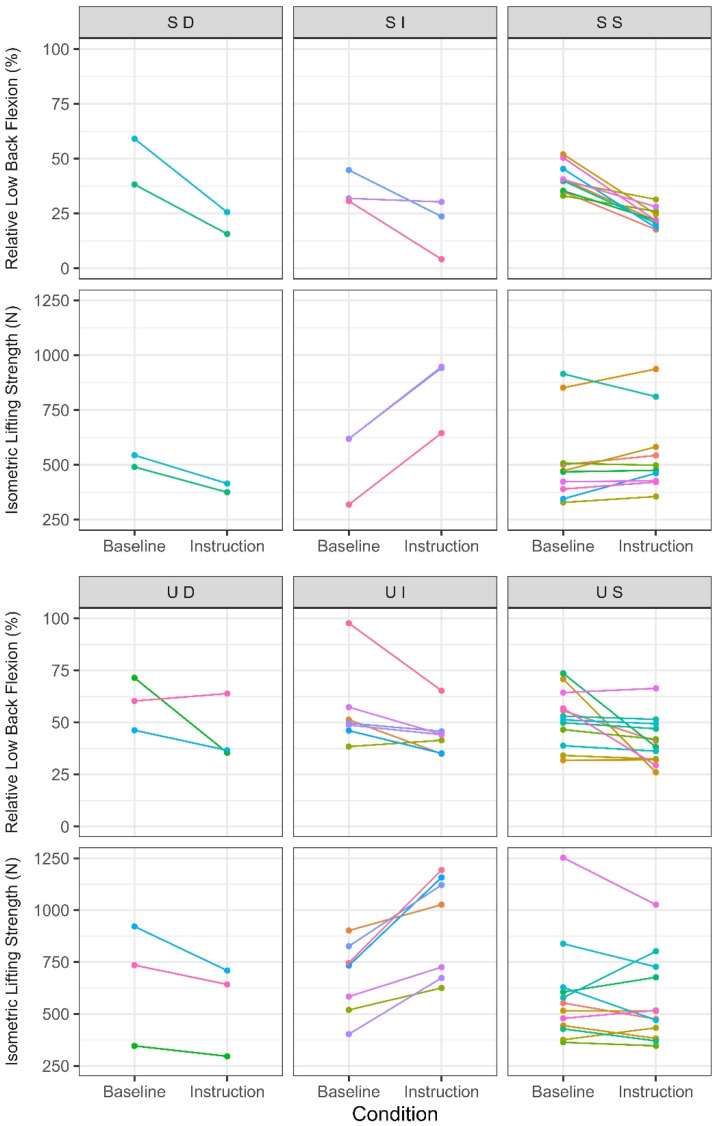
Baseline and instruction condition relative low back flexion (% low back flexion range-of-motion) and isometric lifting strength among those exhibiting greater than 40% flexion range-of-motion during baseline lifting. The first letter of each group corresponds to successful (S) and unsuccessful (U) low back postural responses. The second letter corresponds to isometric lifting strength changes from baseline to instruction, indicating an increase (I), decrease (D) or no change (S). Note that mean values are depicted within each condition, whereas the response classification was based on variation.

Thirty-seven participants (92.5% of total sample) exhibited greater than 40% of their flexion range-of-motion during baseline condition crate lifting based on their personalized confidence interval for low back posture. Within this group, 15 participants (37.5% of total sample) successfully reduced low back flexion in response to the simple verbal instruction, as their personalized low back posture confidence interval was below 40% of their flexion range-of-motion during the instruction condition. Conversely, 22 participants (55% of total sample) were classified as unsuccessful in avoiding low back flexion in response to instruction, as the upper-limit of their confidence interval for low back posture exceeded 40% of their flexion range-of-motion during the instruction condition (Figures 5 and 6).

Strength was variably impacted across all postural groups, indicating that changes in strength occurred independent of changes in low back posture (Figures 5 and 6). Among those who exhibited greater than 40% of their flexion range-of-motion at baseline, isometric lifting strength increased in 6 participants (15% of total sample), decreased in 9 participants (22.5%) and did not change in 22 participants (55%) across both successful and unsuccessful postural response groups.

**Figure 7.**
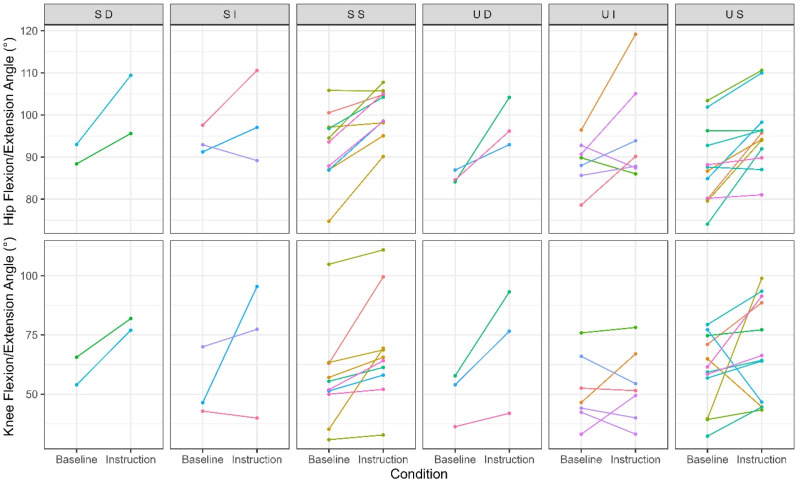
Changes in hip and knee flexion/extension angle for each response group among those exhibiting greater than 40% flexion range-of-motion during baseline lifting. Positive angles indicate flexion and an increase in magnitude indicates an increase in flexion. Note that mean values are depicted within each condition, whereas the response classification was based on variation.

Changes in hip and knee flexion/extension angle varied across both the successful and unsuccessful postural response groups such that some flexed the hip more than the knee and vice versa. This indicates that participants utilized the greater flexibility afforded from specifically cueing low back posture and confirms that participants attempted to change body posture in response to the provided instruction even though they were classified as unsuccessful in avoiding low back flexion (Figure 7).

There appeared to be no association between participant sex and postural response (Successful: 9 females and 6 males, Unsuccessful: 10 females and 12 males, Would not benefit from decreasing low back flexion: 1 female and 2 males) or isometric lifting strength response (Increase: 5 females and 7 males, Decrease: 2 females and 3 males, No Change: 13 females and 10 males).

## Discussion

On average, participants acutely reduced low back flexion while maintaining strength after receiving the simple verbal directive to avoid rounding the low back. However, high heterogeneity in responses was identified which motivated investigating the nuances in the individual responses. Although most participants appeared to maintain (*n* = 23, 57.5% of total sample) or increase (*n* = 8, 20%) strength in response to the low back posture instruction, some participants adopted postures that decreased (*n* = 9, 22.5%) their ability to exert isometric lifting force. These changes in strength did not coincide with changes in low back posture, indicating that the effectiveness of an intervention cannot be completely assessed by solely observing the posture of the body region targeted. Importantly, this investigation identified that simple verbal instruction may be insufficient in controlling low back posture as 22 of the 37 participants that exhibited high low back flexion during baseline lifting (59.5% of 37 and 55% of total sample), did not decrease their low back flexion to be within a range that minimizes passive tissue strain, even though they attempted to change posture. Thus, manual materials handling training requires improved and personalized movement-based interventions to effectively reduce low back injury risk without limiting physical performance potential.

In interpreting the results, it is critical to discuss the limitations that emerged in testing isometric strength of the postures exhibited during crate lifting. Some participants found it difficult to maintain the replicated crate lifting posture when exerting maximal force. This difficulty could in part be attributed to the challenge in producing maximal force in a posture that was adopted to execute a low demand task which did not require high magnitudes of force. Many participants positioned their feet a moderate distance away from the crate. This may have potentially been due to the size of the platform that supported the crate, the area of the force platforms that participants stood on during the experiment or the light mass of the crate that may not have required positioning their body closer to the weight. Due to this distance between the body and crate, and because the handle was not fixed in the vertical direction, some participants would lean back with the handle or be pulled onto their toes as they exerted maximal force (Figure 1(b)), making the task awkward. The novelty of exerting maximal isometric force in the replicated crate lifting posture may have elevated the trial-to-trial variation that was used to classify individual strength responses, masking potentially practically meaningful changes in lifting strength. Since higher variability required larger changes between conditions in order to be classified as an increase or decrease, some strength changes up to an increase of 224N and decrease of 227N were classified as no change (group S, Figures 5 and 6). However, these immediate changes in strength may be meaningful as they exceed changes that typically occur in deadlift strength following 6 weeks of training (approximately 70–190 N increases in 1-repititon maximum depending on training experience (Androulakis-Korakakis et al., 2021; Colquhoun et al., 2018; Zweifel et al., 2017)). The current study administered experimental conditions in a fixed sequence to capture accurate baseline behaviour during crate lifting and to facilitate replication of the crate lifting postures for isometric strength testing. Consequently, some participants may have learned how to adjust and balance their body during the baseline condition so that they were able to apply force more effectively in the instruction condition. However, strength did not increase systematically across trials, and the effect of condition on strength was not dependent on the trial number. Additionally, 9 participants (22.5% of the sample) decreased strength in the instruction condition compared to baseline which suggests that learning or familiarization was not a primary driver of the changes observed. These decreases are unlikely related to fatigue due to the relatively low demand of the mass lifted and the few maximal isometric strength trials performed. Participants were monitored throughout the session and did not report any fatigue. Additionally, previous studies have investigated 56 (Kumar & Garand, 1992) and 90 (Chen et al., 2011) maximum isometric lifting exertions within a single session and did not report observing fatigue. Yet, further investigation is warranted to determine if addressing the abovementioned limitations would alter the results and if different sample populations exhibit different responses.

Although using simple verbal instruction to cue low back posture afforded flexibility for participants to self-organize (Figure 7), over half of the participants (*n* = 22, 55% of total sample; Figures 5 and 6) did not avoid low back flexion as instructed. Using a statistical approach that accounts for heterogeneity in participant responses facilitated this novel insight and this response heterogeneity should be considered when implementing and evaluating movement-based interventions. However, the threshold approach used to categorize low back postural responses does not encompass all biologically meaningful changes. The same evidence showing that passive low back bending stiffness, which can indicate strain on passive tissues (e.g. posterior spinal ligaments), is minimized below 40% of flexion range-of-motion, also suggests that passive stiffness exponentially increases with low back flexion and is highest beyond 80% of flexion range-of-motion (Beach et al., 2005; Buchman-Pearle et al., 2023; Fewster et al., 2021). In the current study, all participants exhibited less than 75% of flexion range-of-motion after instruction, which due to the exponential relationship (McGill et al., 1994), should have had some meaningful effect on passive tissue strain, trunk extensor muscle lines-of-action, and load tolerance of functional spinal units. Yet, some of these participants were classified as unsuccessful in avoiding low back flexion as their instruction condition low back posture confidence interval during crate lifting exceeded 40% of flexion range-of-motion. Additionally, it should be noted that the low and high passive stiffness thresholds mentioned in this discussion (40 and 80% of maximum low back flexion) can vary between individuals (Beach et al., 2005; Buchman-Pearle et al., 2023; Fewster et al., 2021; Zwambag & Brown, 2020). Classification may also change if participants are observed over more trials or longer durations. Thus, it may be beneficial to improve on the current individual response focused approach to better classify changes, both in terms of kinematic and kinetic variables relevant to the magnitude and distribution of low back loading and load tolerance.

Since participants utilized the afforded flexibility but not all participants effectively reduced low back flexion or maintained physical performance potential, manual materials handling training may be enhanced by providing more than simple directives. As opposed to simple verbal instruction, targeting movement features using individualized movement coaching may provide improved efficacy in influencing movement behaviour across all individuals. Based on motor learning principles, offering postural instruction, feedback and training has the potential to be more effective than providing general advice and education (Chan et al., 2022; Kamachi et al., 2021). However, responses may still vary based on the characteristics of the coaching and how it is implemented (Oppici et al., 2021; Pinto et al., 2018, 2024). Prior research has demonstrated that coaching postural features during exercise can positively transfer to unrehearsed nonexercise tasks (Frost et al., 2015). However, providing postural feedback and movement coaching during exercise can also result in individually varying responses (Frost et al., 2015; Pinto et al., 2018), requiring knowledge and strategies to assess and modify motor behaviour on an individual basis. Personalized strategies may equip individuals with the competencies and capacities to coordinate and control their movements in ways that attenuate musculoskeletal loading without hindering physical performance. This aligns with recent rationale to equip workers with the capabilities, opportunities and motivation to self-monitor and -regulate their movement rather than promoting one-size-fits-all directives for safe movement such as the squat lift technique (Denis et al., 2020). The results from the current study further highlight that these personalized coaching strategies should consider effects on both musculoskeletal loading and physical performance potential as changes in isometric lifting strength were independent from changes in low back posture.

## Conclusion

On average, a simple directive targeting low back posture afforded the opportunity to self-organize the rest of the body linkage to maintain physical performance potential. However, it is not guaranteed that every individual will maintain their physical performance potential, as some individuals adopted lifting postures that decreased their isometric lifting strength. Changes in isometric lifting strength did not coincide with changes in low back posture, indicating that the efficacy of the intervention cannot be assessed by solely observing the changes in the postural feature targeted. Although individuals attempted to modify their posture in response to the instruction provided, many were unsuccessful in reducing low back flexion to be within a range that minimizes passive tissue strain with instruction alone. Collectively, results suggest a need for improved and personalized movement coaching to ensure reduction in musculoskeletal loading without hindering physical performance.

## Key points

• Most, but not all, individuals maintained isometric strength when given a simple verbal directive to avoid low back flexion.
• The efficacy of movement-based interventions cannot be completely assessed solely by observing immediate changes in the specific postural feature cued.
• Personalized movement coaching strategies are required as simple verbal directives are insufficient for reducing musculoskeletal loading without hindering physical performance.
